# The Relationship Between Emotion Regulation and Perceptions of Body Image, Health, and Eating in Food Science College Students

**DOI:** 10.3390/ijerph22111636

**Published:** 2025-10-27

**Authors:** Mari Aguilera, Claudia Soar, Ricard Celorio-Sardà, Oriol Comas-Basté, M. Carmen Vidal-Carou, Maria Clara de Moraes Prata Gaspar

**Affiliations:** 1 Departament de Cognició, Desenvolupament i Psicologia de l’Educació, Secció Cognició, Facultat de Psicologia, Campus de Mundet, Universitat de Barcelona (UB), Passeig de la Vall d’Hebron, 171, 08035 Barcelona, Spain; 2 Institut de Neurociències (UBNeuro), Universitat de Barcelona (UB), Passeig de la Vall d’Hebron, 171, 08035 Barcelona, Spain; 3 eHealth Center: Human and Planetary Health Research Centre, Universitat Oberta de Catalunya (UOC), Rambla de Poble Nou 156, 08018 Barcelona, Spain; 4 Nutrition Post-Graduate Program, Department of Nutrition, Federal University of Santa Catarina, Florianopolis 88040900, Brazil; claudia.soar@ufsc.br; 5 Departament de Nutrició, Ciències de l’Alimentació i Gastronomia, Campus de l’Alimentació de Torribera, Universitat de Barcelona (UB), Av. Prat de la Riba 171, 08921 Santa Coloma de Gramenet, Spain; ricard.celorio.97@gmail.com (R.C.-S.); oriolcomas@ub.edu (O.C.-B.); mcvidal@ub.edu (M.C.V.-C.); demoraesprata@ub.edu (M.C.d.M.P.G.); 6 Institut de Recerca en Nutrició i Seguretat Alimentària (INSA·UB), Universitat de Barcelona, Av. Prat de la Riba 171, 08921 Santa Coloma de Gramenet, Spain

**Keywords:** emotional regulation, eating perceptions, body perception, healthy eating

## Abstract

Emotional regulation plays a central role in shaping eating behaviors and body image, though few studies have examined this relationship in students of food sciences. A total of 297 undergraduates from Human Nutrition and Dietetics and Food Science and Technology completed surveys on health, body image, and eating behaviors, along with the Difficulties in Emotion Regulation Scale (DERS). Exploratory factor analysis (EFA) identified four perception dimensions. Pearson correlations and multiple linear regression assessed their relationships with emotional regulation. EFA revealed four factors: (1) health perception, (2) body image and related emotions, (3) weight and diet control, and (4) individual responsibility for diet. No differences emerged by sex or degree. Correlations indicated that three factors were significantly associated with DERS scores, particularly body image and emotions. Multiple regression showed that body image and emotions and weight and diet control significantly predicted emotional regulation, while health perception and individual responsibility were not significant. These findings highlight the role of emotional regulation in shaping perceptions of health and eating, underscoring its relevance in the education of future food professionals. Training food professionals should integrate emotional competencies to support healthier self-perceptions and practices.

## 1. Introduction

The increase in the prevalence of some chronic illnesses such as cardiovascular diseases or cancer as well as the increase in the prevalence of obesity and eating disorders in recent decades has become a serious public health problem [1], exacerbated by the COVID-19 lockdown [2]. In this context, the effects that food consumption can have on human health have become a topic of growing interest both among health and nutrition experts and governments, in addition to becoming a ubiquitous issue in the media and a daily concern for an important part of the population [3]. In order to be able to design and implement strategies that seek to promote healthy eating practices and contexts favorable to the implementation of these practices, it is necessary to improve our understanding of the multiple influences underlying eating behaviors including genetic, economic, environmental, cultural, and social factors as well as their complex interaction [4].

The role of emotions and the capacity to regulate them has emerged as a key factor in understanding eating behavior. The dynamic process of emotional regulation involves two key questions: “What do I feel?” followed by “What do I do with what I feel?”. In this way, the emotion is identified, and its intensity, magnitude, and duration can be controlled [5]. Thus, emotional regulation refers to a human ability to modify the quality and expression of emotions according to individual goals [6,7]. Interestingly, a recent systematic review of studies in children and adolescents suggested that the growing prevalence of overeating behaviors in this population is associated with difficulties in describing emotions, lack of emotional awareness, and expressive suppression strategies (suppression of emotional expression) [8]. Difficulties in emotional regulation have also been reported to influence eating behavior in clinical populations suffering depression, anxiety, and eating disorders such as binge eating, anorexia, or bulimia nervosa. The most frequent maladaptive emotional regulation strategies related to these eating disorders were found to be expressive suppression, avoidance, and rumination [9,10]. Although women are reported to have a higher tendency to develop affective and eating disorders than men, they are also described as having a greater capacity for emotional regulation [11], a paradox that needs further investigation. In this context, emotional regulation plays a central role in how individuals perceive and evaluate their own bodies. From a psychological perspective, body image refers to the subjective representation of one’s body, encompassing perceptual, cognitive, and affective components [12]. Research consistently links low emotional regulation abilities with negative body image and maladaptive eating patterns [13,14,15]. These findings underscore that difficulties in managing emotions may heighten vulnerability to body dissatisfaction and unhealthy eating, a relationship later shaped by broader social norms and cultural ideals.

Health and food professionals, such as dietitians and food scientists and technologists, both individually and collectively, play a central role in the promotion of healthy diets and the transmission of food and nutritional recommendations. However, their personal attitudes, norms, practices, and perceptions regarding food, health, and the body can have a direct impact on their professional activity and recommendations [16]. In a previous study [17], college students of Human Nutrition and Dietetics (HND) and Food Science and Technology (FST) generally perceived their diets to be healthy and believed that food can affect health, but one of the three most important factors preventing them from following a healthy diet was their emotional state. This finding supports another recent study in university students that also reported that the emotional state is one of the main barriers to maintaining a healthy diet [18].

Beyond individual factors, emotions and eating practices are also shaped by social norms and cultural ideals surrounding the body. Society imposes representations and values associated with physical appearance [19], which influence eating behaviors [20]. Lupton [21] refers to it as the “food/health/beauty triplex”. Nowadays, moral attributes of self-control are associated with thinness, making it a symbol of good health, discipline, and beauty as well as an instrument of social mobility [21,22]. What is imposed is truly an ideal, since it escapes reality and is almost unattainable for the majority of people. Body dissatisfaction thus becomes a form of “normality” for many individuals. In Europe, 45% of people report body dissatisfaction [19].

Young adults, especially women, who are in the early years of their professional careers are in a phase of seeking an autonomous life as well as a social and professional identity. The body therefore becomes a form of capital [23], which can affect how individuals are perceived in society. According to Masson [24], the importance of the body in social life was recognized by 78% of French women aged 18 to 24. In the pursuit of being thin, being “on a diet” has become a constant condition for many young women, a phenomenon that Germov and Williams [22] describe as “*the epidemic of dieting women*”.

These dynamics manifest differently across cultures. In Spain, food culture is strongly associated with conviviality, social identity, emotional significance, and collective practices [25,26]. Moreover, although body ideals have become globalized and thinness is valued as a norm of beauty and health, cultural differences still persist in the construction of beauty ideals [19,25]. Previous studies have revealed that the pressure to achieve thinness may be less important in Spain than in other European countries including neighboring ones such as France [19,20]. Nevertheless, these cultural traits coexist with increasing exposure to esthetic ideals emphasizing thinness, body transformation, and self-control. According to a study conducted among university students in Spain, 70% of women wanted to lose weight, and 38.7% had followed a diet during the six months prior to the study [27]. In Spain, the UniHcos Project found that nearly one in five students (19.5%) presented a risk of developing eating disorders, particularly women, with problematic Internet use, binge drinking, and low perceived health emerging as associated factors [28]. Other Spanish studies have also documented strong associations between body dissatisfaction, weight perception, and unhealthy eating patterns [29,30].

Body shape and weight are important concerns and aspects of the dietitian’s socialization and professional identity [31]. Students in the field of nutrition have been described as having a higher tendency to exhibit behaviors related to disordered eating compared with students of other subjects, showing high levels of dietary restraint [32] as well as body dissatisfaction (around 90%) and a desire to be slimmer (83%) [33]. Moreover, it was found that most nutrition students who wanted to lose weight were at high risk of developing behaviors related to problematic eating habits [34]. Studies on these issues conducted with students in the field of food sciences in Spain are still scarce. One study including Spanish dietetics students revealed a widespread situation of body dissatisfaction related to weight [35]. Along this line, nearly half of the food science degree students participating in the study of Gaspar et al. [17] somewhat or strongly agreed that they should control their eating behaviors more, whereas about a third had a troubled relationship with their body and felt guilty when eating.

Despite the evidence that food science students are at higher risk of developing eating disorders, to the best of our knowledge, no studies have focused on assessing their capacity for emotional regulation and its impact on perceived body and health, especially in Spain. Therefore, the main aim of this study was to explore the relationship between emotional regulation and perceptions of body, health, and eating behaviors in HND and FST college students, comparing the capacity for emotional regulation between the two groups. Although both degrees are food-related, they differ in focus and professional orientation: HND centers on health promotion and clinical nutrition, while FST emphasizes the scientific and technological aspects of food production and safety. Therefore, comparing these groups allows for the identification of potential intra-field contrasts in emotional regulation and food-related perceptions among future food professionals. We expected the emotional regulation score to be similar regardless of the degree subject, to increase with years of training, and be higher in women than men. We also hypothesized that a higher capacity for emotional regulation would be associated with a better self-perception of body and health and fewer negative emotions related to eating.

## 2. Materials and Methods

The results presented in this article are part of a broad exploratory and descriptive cross-sectional study based on mixed quantitative and qualitative methodology and carried out between May 2020 and September 2021 by an interdisciplinary team composed of researchers from the fields of anthropology, nutrition, food science, and psychology. The overall aim of this broad study was to analyze food perceptions among college students enrolled in HND and FST bachelor’s degrees. The results presented in this article refer mainly to the relationship between emotional regulation and the self-perception of eating behaviors, body, and health.

The study was conducted in accordance with the Declaration of Helsinki and was approved by the Bioethics Commission of the Universitat de Barcelona.

### 2.1. Participants and Setting

A total of 297 undergraduates (mean age = 21.3 years; 80% women) participated in the study. Participants were recruited in the academic year 2020–2021 through a convenience sampling approach and included students from all academic years (1st to 4th) of the HND and FST bachelor’s degrees at the University of Barcelona. The study employed a cross-sectional design.

As defined by the University of Barcelona, a reference institution in these fields in Spain, the HND degree trains professionals to carry out a variety of food and health-related tasks including the promotion of adequate nutrition according to physiological or pathological needs, and a dietary approach to the treatment and prevention of disease. The FST degree is aimed at training future professionals with the expertise to design and implement methods of producing, packaging, and conserving foods and conduct research into innovations to comply with the current demands for health, safety, and sustainability.

No exclusion criteria were established with respect to participant age, place of residence, or nationality.

### 2.2. Instruments

#### 2.2.1. Emotional Regulation Assessment

The Spanish version of the self-report questionnaire Difficulties in Emotion Regulation Scale (DERS) [36,37] was used to assess emotional regulation. In this instrument, participants are asked to indicate how often the items apply to themselves, with scores ranging from 0 (almost never) to 5 (almost always). A composite DERS score was calculated by adding the scores of all items, higher values indicating more difficulties in emotional regulation. Six subscales can be derived from DERS: awareness and understanding of emotions (awareness), emotional clarity (clarity), level of acceptance of emotions (non-acceptance), level of control of one’s own behavior when experiencing negative emotions (impulse), the ability to maintain goal-directed behaviors when experiencing negative emotions (goals), and the level of access to effective emotional regulation strategies (strategies). In this study, the composite DERS score was used as the primary outcome. The subscales were analyzed exploratorily to examine specific associations with food perception factors.

Good psychometric properties have been reported in the adult version of DERS [37]: Cronbach’s alpha was 0.93 for the composite DERS score, and Cronbach’s alpha internal reliability values for the subscales ranged from α = 0.69 (impulse) to α = 0.90 (non-acceptance). In our sample, a composite DERS score of 0.90 was obtained, and the subscale scores ranged from α = 0.79 (awareness) to α = 0.92 (non-acceptance).

#### 2.2.2. Food Perception Assessment

Given the complexity of analyzing food perceptions [16] and the scarcity of studies in this field [38], a questionnaire was developed specifically for the present study based on initially collected qualitative data as well as previous research on food perceptions of HND college students and/or dietitians [16,28] and the general population [39,40,41]. The questionnaire contained 31 multiple-choice and Likert-scale questions (including those aimed at characterizing the sample) as well as an open-ended question: “With which word do you associate the concept ‘food’? (indicate one word only)”. The original instrument included a first item referring to the participants’ informed consent to take part in the study, which was not considered part of the assessment content. The questionnaire explored a wide range of dimensions related to food perception. Specifically, it included items assessing definitions and meanings of food (e.g., the degree of agreement with statements such as *“Food is any substance that provides nutrients to ensure vital functions”* or *“When you consume food, you are feeding on symbols, myths, and values”*), dietary self-identification with patterns such as *Mediterranean*, *vegetarian/vegan*, or *ecological and natural* diets, and determinants of food choices including factors such as nutritional composition, price, body image concern, or ecological considerations. Other sections address sources of food pleasure (e.g., cooking, sharing meals, controlling food intake), associations with cooking and food sustainability (e.g., health, sustainability, sharing, avoiding food waste, consuming local products), and perceptions of health and body image through items such as *“My diet is healthy”*, *“I should control my diet more”*, or *“I often feel guilty about eating”*. In addition, participants rated different foods—such as meat, fish, vegetables, sweets, and ultra-processed products—according to their perceived taste and healthiness, and expressed their level of confidence in various indicators of food quality (e.g., brand, origin, packaging) and their concern about potential risks including pesticides, additives, animal welfare, and food waste. Items were rated on five-point Likert scales (ranging from *strongly disagree* to *strongly agree*, or *not important* to *very important*), with higher scores reflecting stronger agreement, greater perceived importance, or higher levels of concern, depending on the question. Further details on the study design and sample characteristics and the full questionnaire are available in Gaspar et al. [42].

For the purposes of this study, we selected a subset of 13 items from the broader questionnaire, originally designed to assess multiple dimensions of health, body image, and eating behaviors (item set derived from question 24 in the full instrument). This selection was guided by theoretical considerations, focusing on items most directly related to perceptions of physical health, body image, and eating habits. Specifically, participants rated their agreement with the following statements: “I am in good health”, “My diet is healthy”, “My diet can affect my health”, “It is easy to have a healthy diet”, “Having a healthy diet is a matter of personal will”, “I control my diet”, “I should control my diet more”, “I should do more sport”, “I should lose weight”, “I should gain weight”, “I have a troubled relationship with my body”, “I often feel guilty about eating”, and “My body shape and weight are important factors in my future professional activity”. To empirically validate this item set, an exploratory factor analysis (EFA) was conducted using principal axis factoring with oblique rotation to examine the latent structure and psychometric adequacy of the items. Details of the EFA procedure and results are provided in the Data Analysis and Results sections.

##### Procedure

The food perception and DERS questionnaires were administered online, via the SurveyMonkey website, in April and May 2021. The average time taken to complete the questionnaire was 19 min. To increase adherence and student participation, the questionnaires were administered during class time. In addition, they were sent to all students via emails and WhatsApp groups. A total of 385 responses to the self-report instruments were obtained, 88 of which were excluded because they were incomplete, leaving 297 complete responses (77.1%). Responses were considered complete when both instruments used in the study were filled out.

##### Data Analysis

Descriptive statistics (means, standard deviations, and frequencies) were calculated for all study variables. The Student’s t-test was used when two groups were compared, ANOVA when comparing more than two groups, and the chi-square test when categorical variables were compared. To identify underlying dimensions of the students’ perceptions regarding health, body, and eating behaviors, an exploratory factor analysis (EFA) with principal axis factoring and oblique rotation was performed. The number of factors was determined based on eigenvalues > 1, scree plot inspection, and theoretical interpretability. Sampling adequacy was assessed with the Kaiser–Meyer–Olkin (KMO) statistic and Bartlett’s test of sphericity.

Bivariate relationships between the derived perception factors and emotion regulation scores were initially assessed using Pearson correlations. Although the DERS comprises several subscales, the composite score was selected as the primary outcome for the main regression analyses, as it provides a reliable index of overall emotional regulation difficulties and reduces the risk of inflated Type I error. In addition, a series of exploratory multiple regression models were conducted using each DERS subscale (awareness, impulse, non-acceptance, goals, clarity, and strategies) as outcomes to examine whether the observed associations generalized across specific domains of emotion regulation. Prior to the regression analyses, assumptions of multiple linear regression were examined. Independence of residuals was examined using the Durbin–Watson test, multicollinearity was evaluated via VIF, normality of residuals was assessed with the Shapiro–Wilk test and Q–Q plots, and linearity and homoscedasticity were inspected through residual scatterplots. Statistical significance was set at *p* < 0.05. All analyses were conducted using IBM SPSS 28 and Jamovi 2.6.44.

## 3. Results

Out of the 297 participants, 150 were studying HND and 147 FST, representing 46.7% and 44.5% of the total enrolments in the two degrees, respectively. The overall mean age was 21.25 (±3.16) years (21.76 ± 3.79 for HND students and 20.73 ± 2.24 for FST students). Most informants were female (239, 80%).

### 3.1. Emotional Regulation Among College Students of Food Science

No significant differences were found between HND and FST students in the total or subscale scores of the DERS questionnaire (Table 1). When comparing students of different years, those in the 4th year tended to exhibit better emotional regulation compared with 1st or 2nd/3rd year students according to all the DERS variables and the total DERS score, although the trend was not statistically significant (Table 2). Additionally, as shown in Table 3, no statistically significant differences in total or subscale scores were observed between men and women.

### 3.2. Emotional Regulation and Self-Perception of Body, Health and Eating Behavior

An exploratory factor analysis (EFA) was conducted using the Kaiser criterion (eigenvalues above 1) as the extraction method, which resulted in a four-factor solution. The sampling adequacy was assessed using the Kaiser–Meyer–Olkin (KMO) measure, yielding a value of 0.705, indicating a moderate level of adequacy for factor analysis. Bartlett’s test of sphericity was statistically significant (χ^2^(78) = 825.972, *p* < 0.001), suggesting that the correlation matrix was not an identity matrix and that the data were suitable for factor analysis. Additionally, the Chi-squared test for model fit yielded a significant result (χ^2^(32) = 79.308, *p* < 0.001), indicating that the extracted factor structure adequately represented the data. Factor 1 (perception of health) included six items (I am in good health, My diet is healthy, It is easy to have a healthy diet, I control my diet, I should control my diet, I should do more sport), communalities ranged from −0.85 to 0.77. Factor 2 (perception of body image and related emotions) included two items (I have a troubled relationship with my body, I often feel guilty about eating), communalities above 0.6. Factor 3 (perception of body weight) included two items (I should lose weight, I should gain weight) with communalities below −0.48. Finally, Factor 4 (diet individual responsibility perception), had 1 item (Having a healthy diet is a matter of personal will), communality = 0.58. Two items were excluded from the factor analysis due to low factor loadings (<0.40), indicating that they did not strongly contribute to any of the identified factors (My diet can affect my health, My body shape and weight are important factors in my future professional activity).

As can be seen in Table 4, when analyzing the relationship between four factor solution and emotional regulation variables, perception of health (Factor 1) showed a weak negative correlation with the composite DERS score. In other words, a better capacity for emotional regulation was associated with more positive perceptions of health status. Similarly, perception of health (Factor 1) presented a weak negative correlation with strategies and impulse, with the strongest negative correlations observed with awareness. Thus, individuals with greater emotional awareness, who reported a lower tendency to feel out of control, tended to have a better perception of their own health, as did those with a more effective use of emotional regulation strategies.

Perception of body image and related emotions (Factor 2) was positively correlated with the composite DERS score (medium effect size). Moreover, significant correlations were observed with five of the six subscales but with small to moderate effect sizes. Therefore, poorer overall emotional regulation as well as specific skills regarding awareness, non-acceptance, impulse, goals, and strategies were associated with dissatisfaction with body shape and a higher tendency to experience negative food-related emotions such as feeling guilty. Factor 3, perception of body weight, did not show significant associations with any of the analyzed variables related with emotional regulation.

Finally, difficulty in emotional regulation indicated by higher composite DERS scores had a weak correlation with diet concept perception, Factor 4. Among the subscales, this perception was significantly associated with lower awareness, acceptance, clarity of emotions, and less impulse control, but with small effect sizes.

A multiple linear regression examining the contribution of all four factors to the composite DERS score revealed that the overall model was significant (F(4, 292) = 12.7, *p* < 0.001) and explained 13.6% of the variance (adjusted R^2^ = 0.136). Among the predictors, Factor 2 (body image and emotions) was the strongest significant predictor (β = 10.022, *p* < 0.001), and Factor 3 (weight and diet control) also contributed significantly (β = −5.130, *p* = 0.002). Factors 1 and 4 were not significant (*p* > 0.05). Prior to interpretation, model assumptions were checked. The independence of residuals was supported by the Durbin–Watson test (DW = 1.91, *p* = 0.428), indicating no significant autocorrelation. Collinearity diagnostics confirmed the absence of multicollinearity (VIF = 1.26–1.54). The Shapiro–Wilk test suggested a deviation from normality (*p* < 0.001), but inspection of the Q–Q plot showed residuals to be approximately normally distributed, with only minor deviations at the extremes. Residual scatterplots against all predictors revealed random distributions around zero without systematic patterns or heteroscedasticity, supporting the assumptions of linearity and homoscedasticity.

Exploratory regression models were conducted for each DERS subscale to examine. For awareness, the overall model was significant, F(4, 292) = 5.60, *p* < 0.001, accounting for 7.1% of the variance; perception of body image and related emotions (Factor 2) was the only significant predictor (β = 0.64, *p* = 0.021). For impulse, the model explained 11.3% of the variance, F(4, 292) = 9.31, *p* < 0.001; Factor 2 showed a strong positive effect (β = 1.89, *p* < 0.001), while perception of body weight (Factor 3) was negatively associated (β = −1.28, *p* = 0.001). For non-acceptance, the model explained 12.8% of the variance, F(4, 292) = 10.7, *p* < 0.001, with Factor 2 positively (β = 3.34, *p* < 0.001) and Factor 3 negatively (β = −1.40, *p* = 0.015) associated with difficulties. For goals, the model reached significance, F(4, 292) = 3.95, *p* = 0.004, with 5.1% of the variance explained; again, only Factor 2 emerged as a predictor (β = 1.25, *p* < 0.001). For clarity, the model accounted for 6.3% of the variance, F(4, 292) = 4.87, *p* < 0.001, with Factor 2 positively (β = 1.06, *p* = 0.001) and Factor 3 negatively (β = −0.58, *p* = 0.047) predicting difficulties. Finally, for strategies, the regression was significant, F(4, 292) = 10.5, *p* < 0.001, explaining 12.6% of the variance, with Factor 2 as a strong positive predictor (β = 1.83, *p* < 0.001) and Factor 3 as a negative predictor (β = −0.93, *p* = 0.005). Across models, perception of health (Factor 1) and perception of individual responsibility for diet (Factor 4) did not emerge as significant predictors. After applying Bonferroni correction for multiple comparisons (α = 0.0083), Factor 2 remained a consistent predictor across all subscales (all *p*s < 0.001). In contrast, the negative associations of Factor 3 with non-acceptance (*p* = 0.015) and clarity (*p* = 0.047) no longer reached the adjusted threshold, while its effects on strategies (*p* = 0.005) and impulse (*p* = 0.001) remained significant.

## 4. Discussion

The capacity for emotional regulation was found to be similar among the HND and FST students, and tended to improve over the years of training, although without statistical significance. As this study followed a cross-sectional design, these differences reflect cohort variations rather than within-individual changes. This improvement could be partly due to the maturation process in the late phase of adolescence, and the importance of emotional regulation when adapting to social life at university [43]. No differences in levels of emotional regulation were observed between women and men. This finding contrasts with previous research suggesting that women tend to use a wider variety of strategies and apply them more flexibly across contexts [44]. One possible interpretation is that, in our sample, the influence of gender-related social expectations during late adolescence may have affected both groups similarly, reducing the expression of potential differences typically observed in other populations [45].

Food science students with better emotional regulation tended to report a more positive perception of their health. This factor integrates several interrelated aspects such as overall health status, adherence to a healthy diet, dietary control, and the need for physical activity. These results are consistent with those of Isasi et al. (2013) [46], who found that better emotional regulation was related to healthy lifestyle behaviors in adolescents such as eating healthier food (fruit and vegetables) and physical activity. Moreover, previous research has related emotional regulation with an ability to control overeating, low emotional awareness being associated with overeating dysregulation when food was readily available [47]. Additionally, loss of control of eating has been linked with maladaptive emotional strategies such as withdrawal or rumination, whereas physical activity has been proposed as a beneficial means of overcoming difficulties in emotional regulation [48,49]. However, when considering all perceptual dimensions simultaneously, this association did not remain significant, suggesting that its apparent influence on emotional regulation may be largely explained by body-related emotions.

The perception of body image and related emotions factor showed the strongest and most consistent association with emotion regulation, both at the global level and across nearly all subscales of the DERS. This highlights the pervasive influence of negative body-related emotions—such as guilt or dissatisfaction—on regulatory functioning. A negative perception of one’s body may contribute to difficulties in accepting emotional experiences, setting and pursuing goals when emotionally distressed, and accessing effective regulation strategies as well as reduced emotional awareness and impaired impulse control. Notably, as reported by Gaspar et al. [17], 30% of the food science students declared some degree of body dissatisfaction and a desire to lose weight. This relationship appeared to be particularly relevant among nutrition students, who have been found to exhibit a higher tendency toward disordered eating behaviors compared with students from other fields [32,33,34]. Furthermore, previous studies have found that dietetics students are influenced by social norms promoting thinness, associating being thin with having greater professional credibility, and that similar pressure is experienced by dietetics professionals [38,50]. Studies indicate that dietitians tend to perceive people with obesity as less competent and express negative attitudes toward them [51], whereas dietitians with obesity report feeling discriminated against and believe their weight limits their professional activity [52].

In contrast, the perception of body weight factor showed a weaker and less consistent relationship with emotion regulation. While correlations suggested little association, multivariate analyses indicated that concerns about body weight may, in some cases, relate to fewer difficulties in domains such as impulse control and the use of strategies. This pattern could reflect the role of weight monitoring as a form of external behavioral regulation, which may provide a sense of control but does not necessarily translate into adaptive emotional competencies [9].

Perception of individual responsibility for diet (Factor 4) was initially correlated with poorer emotion regulation, particularly reduced awareness, acceptance, clarity, and impulse control. However, these associations did not remain significant once the other perceptual dimensions were considered, suggesting that responsibility narratives may overlap with body-related emotions rather than exerting an independent effect. The emphasis on individualizing dietary practices is a characteristic of the process of the medicalization of food that overlooks the broader social, political, and economic dimensions of eating practices, placing the burden solely on the individual [3].

Overall, these findings suggest that body-related perceptions—and particularly their emotional load—are the key determinants of emotional regulation in this student population, whereas general health beliefs and individual responsibility narratives appear less influential when considered alongside them. In Western industrialized countries, hegemonic esthetic standards rooted in the valorization of thinness reinforce these responsibility discourses, repeatedly conveying the idea that eating is an act of willpower and that both diet and body shape depend on individual self-control [53]. Such individualization of dietary practices not only obscures social and economic dimensions, but also intensifies feelings of guilt, as observed among students who internalize these external pressures. To address these issues and reduce the importance given to body shape, food science curricula should integrate the emotional aspects of eating behaviors and explicitly address the psychological and social factors underlying body attitudes, esthetic norms, food, and health [54].

This study had some limitations. The sample was restricted to college students of HND and FST degrees at the Universitat de Barcelona, with about 70% of participants resident in the Barcelona province. Therefore, although this university is one of the biggest in Spain, other universities should be included in future studies to generalize the results. Moreover, it could be relevant, in future research, to compare the data from this sample of food science students with students from other fields of knowledge. Additionally, only one instrument was used to measure emotional regulation. A more comprehensive assessment would include, for example, measures to capture emotional regulation strategies such as emotional suppression and qualitative techniques such as semi-structured interviews. Moreover, another limitation of this study was the use of a 13-item subset from a broader ad hoc questionnaire. Although the selection was theory-driven and supported by exploratory factor analysis, the reduced item set may not have fully captured the multidimensionality of health, body image, and eating perceptions. Future studies should validate the full instrument or employ standardized measures to confirm and extend these findings. Furthermore, the overrepresentation of women in the sample could affect the ability to detect differences in emotional regulation compared with men. Nevertheless, this gender imbalance is characteristic of food science degrees, especially Human Nutrition and Dietetics [55,56].

## 5. Conclusions

This study contributes new evidence on the emotional underpinnings of food- and body-related perceptions among students in food sciences, a population that has been little studied in Spain but which plays a crucial role in shaping future dietary behaviors and the public understanding of food and nutrition. Our findings indicate that students in both HND and FST programs show comparable levels of emotional regulation. However, difficulties in emotional regulation were strongly linked to negative perceptions of body image and food-related emotions, suggesting that emotional factors, rather than cognitive beliefs about health or personal responsibility, most strongly influence how these students perceive their own bodies and eating habits.

These results highlight that developing professional competence in the food sciences requires more than technical knowledge, and also involves cultivating emotional awareness, self-acceptance, and reflective skills regarding one’s own relationship with food and the body. Integrating the psychological and emotional dimensions of eating into university curricula could foster healthier self-perceptions among students and ultimately enhance their ability to communicate balanced, empathetic, and effective health messages to the public. Likewise, it would be important to incorporate more approaches from the social sciences on the social construction of body norms and representations, promoting a more critical perspective on this phenomenon.

By embedding emotional and social intelligence training and discussions on body image and food culture into educational frameworks, higher education institutions can help prepare future food professionals who are not only scientifically competent, but are also emotionally resilient, socially sensitive, and capable of promoting a more holistic and human-centered vision of healthy eating.

## Figures and Tables

**Table 1:** Emotional regulation in food science college students.

DERS Subscales	HND (*n* = 150)	FST (*n* = 147)	Comparison	
	Mean (SD)	Mean (SD)	*t*	*p* Value
Awareness	9.57 (3.35)	9.69 (3.50)	−0.30	0.762
Impulse	10.03 (4.59)	9.99 (4.66)	0.09	0.930
Non-acceptance	15.78 (7.18)	15.12 (6.89)	0.81	0.421
Goals	12.03 (3.87)	12.03 (4.49)	−0.01	0.999
Clarity	9.65 (3.54)	9.63 (3.46)	0.05	0.959
Strategies	8.42 (4.07)	8.59 (4.01)	−0.37	0.714
Composite DERS	65.49 (20.49)	65.06 (20.56)	0.18	0.856

Note: HND: Human Nutrition and Dietetics; FST: Food Science and Technology; DERS: Difficulties in Emotion Regulation Scale.

**Table 2:** Emotional regulation in food science college students of all years (1st, 2nd/3rd, and 4th).

DERS Subscales	1st (*n* = 95)	2nd & 3rd (*n* = 119)	4th (*n* = 83)	Comparison	
	Mean (SD)	Mean (SD)	Mean (SD)	f	*p* Value
Awareness	9.27 (3.47)	9.94 (3.41)	9.60 (3.39)	1.01	0.365
Impulse	9.99 (4.56)	10.35 (4.69)	9.55 (4.59)	0.72	0.490
Non-acceptance	15.27 (6.39)	15.64 (7.42)	15.39 (7.24)	0.07	0.928
Goals	12.08 (4.03)	12.53 (4.09)	11.26 (4.40)	2.27	0.106
Clarity	9.82 (3.45)	9.89 (3.50)	9.07 (3.51)	1.56	0.213
Strategies	8.82 (4.09)	8.56 (3.83)	8.07 (4.25)	0.78	0.461
Composite DERS	65.26 (19.52)	66.91 (20.27)	62.96 (21.86)	0.91	0.406

Note: DERS: Difficulties in Emotion Regulation Scale.

**Table 3:** Emotional regulation in women and men food science college students.

DERS Subscales	Women (*n* = 239)	Men (*n* = 57)	Comparison	
	Mean (SD)	Mean (SD)	t	*p* Value
Awareness	9.64 (3.48)	9.56 (3.24)	0.16	0.870
Impulse	9.99 (4.63)	9.93 (4.44)	0.09	0.932
Non-acceptance	15.41 (7.01)	15.63 (7.01)	−0.21	0.835
Goals	12.01 (4.16)	12.02 (4.26)	−0.01	0.994
Clarity	9.67 (3.53)	9.35 (3.09)	0.63	0.532
Strategies	8.54 (4.14)	8.28 (3.57)	0.47	0.640
Composite DERS	65.26 (20.62)	64.77 (19.84)	0.16	0.871

Note: DERS: Difficulties in Emotion Regulation Scale. One participant did not provide this information.

**Table 4:** Pearson correlations between the emotional regulation variables and the four-factor concerning perceptions of body, health, and eating behavior among food science college students.

Variables (*n* = 297)	1	2	3	4	5	6	7	8	9	10	11
1. FA1—Health perception	—										
2. FA2—Body image and emotions	−0.31 ***	—									
3. FA3—Body weight	−0.04	0.51 ***	—								
4. FA4—Individual responsibility	0.44 ***	−0.17 **	0.09	—							
5. Awareness	−0.21 ***	0.17**	−0.00	−0.19 **	—						
6. Non-acceptance	−0.09	0.32***	0.04	−0.12 *	0.34 ***	—					
7. Goals	−0.08	0.20***	0.02	−0.01	0.17 **	0.44 ***	—				
8. Clarity	−0.09	0.20***	−0.01	−0.13 *	0.48 ***	0.50 ***	0.39 ***	—			
9. Strategies	−0.16 **	0.31***	0.02	−0.04	0.30 ***	0.67 ***	0.62 ***	0.52 ***	—		
10. Impulse	−0.16 **	0.26 ***	−0.04	−0.14 *	0.33 ***	0.60 ***	0.59 ***	0.55 ***	0.70 ***	—	
11. Composite DERS	−0.16 **	0.33 ***	0.01	−0.14 *	0.53 ***	0.84 ***	0.70 ***	0.73 ***	0.85 ***	0.84 ***	—

Note. Pearson correlations reported. *p* < 0.05 *, *p* < 0.01 **, *p* < 0.001 ***.

## Data Availability

Data will be made available upon request by email to the first author.
